# Soluble Fms-like tyrosine kinase-1 polymorphisms associated with severe-spectrum hypertensive disorders of pregnancy

**DOI:** 10.1007/s00404-024-07917-0

**Published:** 2025-01-13

**Authors:** Tracy Chen, Claire E. Baldauf, Kevin S. Gill, Sue Ann Ingles, Trevor A. Pickering, Melissa L. Wilson

**Affiliations:** 1 Department of Population and Public Health Sciences, Keck School of Medicine, University of Southern California, Los Angeles, CA 90089 USA; 2 Fetal and Neonatal Institute, Division of Neonatology, Department of Pediatrics, Keck School of Medicine, Children’s Hospital Los Angeles, University of Southern California, Los Angeles, CA USA

**Keywords:** Soluble fms-like tyrosine kinase-1, Polymorphism, Hypertensive disorders of pregnancy, Genetics, Dyads, Triads

## Abstract

**Background:**

sFLT-1 has been implicated in the pathogenesis of HDP. We aimed to examine the role of maternal and fetal polymorphisms in risk of HDP and severe-spectrum disease.

**Methods:**

Cases of HDP (143) and controls (169) from mother-baby dyads were recruited at the Los Angeles County Women’s and Children’s Hospital (WCH). Cases of severe disease (99) and controls (31) from mother-father-baby triads were recruited through HELLP syndrome websites. Four sFLT-1 SNPs (rs7993594, rs3751395, rs7983774, and rs664393) were genotyped. Data was analyzed using a log-linear regression model in the Haplin package in R.

**Results:**

Maternal double dose of the A allele (rs7993594) exhibited a nominally significant increased risk of HDP (RR = 3.52, 95% CI 1.08, 11.20). In the severe-spectrum cohort, a marginally significant protective effect among mothers carrying infants with a single dose of the A allele (rs7993594) was observed (RR = 0.59, 95% CI 0.36, 0.98) and double-dose maternal carriage of the G-t-G-G haplotype increased risk of severe disease (RR = 4.13, 95% CI 1.22, 13.80).

**Conclusion:**

The maternal rs7993594 A allele appears to be associated with increased risk of HDP. Double-dose maternal carriage of the G-t-G-G haplotype increased risk of severe disease whereas the fetal rs7983774 A allele appears to be associated with decreased risk.

## Take home message

**Table:** 

Maternal sFLT-1 rs7993594 genotype appears to be associated with an increased risk of HDP, while the fetal sFLT-1 rs7983774 appears to be associated	

## Introduction

Hypertensive disorders of pregnancy (HDP) can complicate up to 10% of pregnancies in the United States [25] and encompass a range of severities, including preeclampsia (PE), eclampsia, chronic hypertension with superimposed preeclampsia, and gestational hypertension, as defined by the American College of Obstetricians and Gynecologists (ACOG) (Hypertension, 2013). Maternal complications at time of delivery include placental abruption, heart failure, intravascular coagulopathy, acute renal failure, and pleural effusions [18, 37]. Long term outcomes of maternal HDP consist of increased risks of cardiovascular disease (CVD), diabetes mellitus, chronic kidney disease, and stroke [9, 10, 35]. Neonatal complications include fetal growth restriction, intrapartum death, and stillbirth with long-term associations seen in childhood asthma, early-onset CVD, and diabetes mellitus within the first decade of life [14, 16, 40].

HDP comprises a variety of clinical manifestations, and the molecular etiology remains largely unclear. Studies have implicated an imbalance in the circulating plasma concentration of angiogenic and anti-angiogenic factors to be integral in the pathophysiology of PE [7, 33]. The upregulation of the anti-angiogenic protein, soluble fms-like tyrosine kinase-1 (sFLT-1), and the reduction of angiogenic factors, placental growth factor (PlGF) and vascular endothelial growth factor (VEGF), have been associated with the development of PE [19, 20, 32].

Located on Chromosome 13, sFLT-1 is a splice variant of the VEGF receptor-1 (FLT-1) that inhibits angiogenesis by binding the pro-angiogenic factors PlGF and VEGF. Studies have revealed excess sFLT-1 levels and reduced circulating free PlGF and VEGF in women before the onset of PE [19, 20] and in preeclamptic placentas, with elevated levels usually subsiding after placenta delivery [23]. An imbalance in angiogenic factors is believed to lead to adverse pregnancy outcomes [2] such as the widespread endothelial dysfunction that is characteristic of PE. As reviewed by Demir et al., PlGF and VEGF regulate the growth and differentiation of trophoblasts, promote angiogenesis, and remodeling of the spiral arteries during normal placental development [5]. The release of excess sFLT-1 can be attributed to placental oxidative stress, thus preventing the angiogenic factors, VEGF and PlGF, from binding to their receptors and initiating angiogenesis [1]. Genetic association studies have revealed relationships between some sFLT-1 SNPs and risk for PE [17, 24], though conflicting results have been observed in different populations [22, 29].

The primary aim of this study is to evaluate whether four polymorphisms ( rs7993594, rs3751395, rs7983774, and rs664393) and haplotypes within the FLT1 gene are associated with pregnancies complicated by HDP and severe PE/HELLP syndrome in mother-baby dyads and mother-father-baby triads, respectively. We also examine the presence of any parent-of-origin effects.

## Materials and methods

### Editorial policies and ethical considerations

This study has received ethical approval from the Institutional Review Board (HS-06–00111). Complete written informed consent was obtained from patients for the purposes of this study.

### Subjects

HDP: This study population consisted of mother-baby dyads of cases of HDP (n = 143) and healthy controls (n = 169) retrospectively recruited from Los Angeles County (LAC) + University of Southern California (USC) Women’s and Children’s Hospital (WCH), identified through delivery logs in 1999–2006 and during postpartum hospital stays in 2007–2008. Cases recruited from WCH were women diagnosed with PE, eclampsia, gestational hypertension (GH), or HELLP syndrome, identified by the attending physician and confirmed via chart review. Controls from this same population were women with an absence of HDP clinical diagnosis in pregnancy.

Severe PE/HELLP: Mother-father-baby triads (n = 130) were included in this study population. Participants were recruited online through two HELLP syndrome-centered research websites (www.hellpsyndromesociety.org or https://www.facebook.com/pages/Hellp-Syndrome-Research-at-USC/163745723652843). Cases (n = 99 triads) were women with self-identified HELLP syndrome, verified through medical record abstraction whenever possible (92.9%). Controls (n = 31 triads) were friends of cases who delivered a baby within 5 years of the index pregnancy and self-reported that they did not experience HDP during their pregnancy.

### Case definition

HDP: Cases were defined as PE if they had a systolic blood pressure ≥ 140 and/or diastolic BP ≥ 90 on two occasions at least 6 h apart and proteinuria, as demonstrated by ≥  + 1 on a dipstick or ≥ 300 mg/dL/24 h [15]. Some cases were identified via the delivery log records, which contain information regarding all events during labor and delivery. Contact information was provided to the study team and a research coordinator contacted them to determine interest and appropriateness for the study. As this method was inefficient, most cases were identified at the time of delivery via communication with attendings. Participants with hypertension but no proteinuria were defined as gestational hypertensives. Since we found no differences between these groups, they were combined for analysis.

Severe PE/HELLP: Cases were verified to be HELLP syndrome if they met the following criteria: (1) Hemolysis, as evidenced by peripheral blood smear indicating abnormal red blood cells or by LDH ≥ 600, (2) AST or ALT ≥ 70, and (3) Platelets < 100 K with or without hypertension and proteinuria. Women meeting 2 of 3 criteria were classified as severe PE, as all presented with significant hypertension (BP ≥ 160/110 mmHg on two separate occasions, at least 6 h apart) and proteinuria (500 mg/dL/24 h or + 3 dipstick on two occasions at least 6 h apart). Cases for whom records could not be obtained were classified as severe PE (n = 6, 6.1%), as all study participants reviewed met at least these criteria.

### Questionnaire

Participant data was obtained through a standardized risk questionnaire in English or Spanish to suit the Los Angeles Hispanic community (HDP population) based on the original developed at the University of Pittsburgh (R. Ness, personal communication). The same questionnaire, in English, was also used for the severe PE/HELLP population. Details regarding family history, medical history, reproductive and sexual history, and the affected pregnancy were collected via chart abstraction.

### Chart abstraction

Medical records were requested from the hospital of delivery for all cases as well as controls for the HDP population only. In the severe PE/HELLP population, obstetric and hospital records were requested from the treating obstetrician and delivery hospital. To verify diagnosis, records were reviewed by one of the investigators (MLW). A standardized data abstraction form, with information regarding prenatal visits, delivery, obstetric history, and comorbidities, were used to abstract record data.

### Selection of polymorphisms

Four SNPs were selected for analysis within the FLT1 gene, intended to capture the majority of the variation in the gene: rs7993594, rs3751395, rs7983774, and rs664393. Based upon a search conducted in 2008, each SNP was selected if it met at least one of the following criteria: 1) associated with health outcomes in one or more peer-reviewed literature, 2) known to be a functional polymorphism based on existing publications, 3) located in a coding region, resulting in a non-synonymous amino acid substitution, 4) located in a regulatory or non-coding region, or 5) located in an intronic region of the gene. SNPs were evaluated with respect to their linkage disequilibrium, however, we did not specifically select tag SNPs. Details on the selected SNPs are displayed in Tables 1 and 2 [27].

**Table 1:** FLT1 single nucleotide polymorphisms

SNP (rs#)	Variant type	Position	Consequence	Allele	Reference Allele		Alternate Allele		Associations
					LA^1^	EU^2^	LA	EU	
rs7993594	SNV	chr13:28,497,814 (GRCh38.p14)	None	C > T	C = 0.89	C = 0.76	T = 0.11	T = 0.24	None
rs3751395	SNV	chr13:28,384,818 (GRCh38.p14)	FLT1: Intron Variant	C > A	C = 0.28	C = 0.55	A = 0.72	A = 0.45	None
rs7983774	SNV	chr13:28,390,188 (GRCh38.p14)	FLT1: Intron Variant	G > A	G = 0.89	G = 0.72	A = 0.11	A = 0.28	TP-53 mutated rectal tumors [38]
rs664393	SNV	chr13:28,496,864 (GRCh38.p14)	FLT1: 5’ UTR	T > C	T = 0.10	T = 0.11	C = 0.90	C = 0.89	Hepatocellular carcinoma (HCC) [34]

^1^Latin American population

^2^European population

Source: NCBI dbSNP (NCBI, 2019)

**Table 2:** Pairwise linkage disequilibrium in FLT1 single nucleotide polymorphisms^1^

Pairwise SNP (rs#)	Pairwise linkage disequilibrium (r2, d)
rs7993594-rs3751395	(0.035, 0.394)
rs7993594-rs7983774	(0.128, 0.383)
rs7993594-rs664393	(0.041, 1)
rs3751395-rs7983774	(0.151, 0.771)
rs3751395-rs664393	(0.051, 0.775)
rs7983774-rs664393	(0.13, 0.625)

^1^Calculated from 1000 Genomes Project population

Source: LDlinkR Haplin package [26]

### Sample collection for analytic population

DNA samples for the HDP population were obtained via saliva in mouthwash (73% of samples) or buccal swabs for mothers and infants. For the severe PE/HELLP syndrome population, DNA samples (n = 367) were collected via buccal swabs (n = 96) or saliva samples (n = 271) (DNA Genotek, Ottawa, Canada). The DNA sampling method did not affect genotyping failure rates. Buccal swab samples were extracted using QIAmp DNA mini kits, according to the manufacturer's protocol (Qiagen, Valencia, CA). Samples obtained from saliva were extracted using ethanol precipitation and following manufacturer protocol for those collected via Oragene saliva kits (DNA Genotek, Ottawa, Canada). For all SNPs, we repeated 5% of samples to assess accuracy of the results. We did not observe any inconsistencies. Genotyping failure rate for each SNP was as follows: (1) rs7993594: 0%, (2) rs3751395: 0.98%, (3) rs7983774: 0.16% and (4) rs664393: 0.33%.

### Statistical analysis

Maternal demographic and clinical characteristics are presented as frequencies and percentages for categorical variables or means ± standard deviations or as median (IQR) for continuous variables, stratified by case–control status for each patient cohort (Table 3). The Haplin package (Version 7.3.0) implemented in the R statistical programming language was used to analyze data from mother-baby dyads in the HDP population and mother-father-baby triads in the HELLP syndrome population (R Foundation for Statistical Programming, Vienna, Austria). In order to address correlation between familial genotypes, we have used a log-linear regression model that also allows for examination of parent-of-origin effects [11, 39]. Haplin is a flexible and robust software used to perform genetic association analyses of case-parent triad data using a log-linear method, estimating single- and double-dose relative risks (RR) and 95% confidence intervals for each genotype and haplotype (Gjessing et al., 2006). The most frequent alleles and haplotypes are used as reference. Haplin evaluates free response models, parent-of-origin effects, and derives likelihood estimation tests of association. Unknown phases of haplotypes or missing genotypic data (father or child) were imputed using Haplin’s Expectation–Maximization (EM) algorithm [11].

**Table 3:** Maternal demographics and clinical characteristics stratified by case–control status

	HDP				SEVERE PE/HELLP SYNDROME			
Variable	N	Cases (n = 143)	N	Controls (n = 169)	N	Cases (n = 99)	N	Controls (n = 31)
Age	142	27.9 ± 7.4	169	26.8 ± 7.0	98	31.0 ± 4.1	27	32.1 ± 4.0
Hispanic race (%)	142	138 (97.2)	169	164 (97.0)	–	NA	–	NA
White race (%)	–	NA	–	NA	98	97 (99.0)	28	28 (100.0)
Gestational age at delivery (weeks)	142	36.8 ± 3.3	168	38.7 ± 2.0	96	33.1 ± 4.1	20	39.6 ± 1.8
Pre-pregnancy weight (lbs)	143	151.9 ± 36.0	169	140.0 ± 27.1	81	147.5 ± 32.0	–	NA
Max systolic blood pressure (mmHg)	134	162.9 ± 15.7	159	117.9 ± 10.9	92	159.6 ± 22.0	–	NA
Max diastolic blood pressure (mmHg)	134	97.4 ± 9.9	159	68.9 ± 9.0	92	97.6 ± 13.4	–	NA
Nulliparity (%)	142		168		96		23	
Nulliparous		61 (43.0)		52 (31.0)		83 (86.5)		12 (52.2)
Parous		81 (57.0)		116 (69.0)		13 (13.4)		11 (47.8)
Gravidity (%)	142		169		92		21	
1		50 (35.2)		43 (25.4)		68 (73.9)		10 (47.6)
2		34 (23.9)		48 (29.0)		16 (17.4)		6 (28.6)
3		20 (14.1)		30 (17.8)		5 (5.4)		3 (14.3)
4 or more		38 (26.8)		47 (27.8)		3 (3.3)		2 (9.5)
Status	–		–		99		–	
Severe PE		NA		NA		56 (56.6)		NA
HELLP		NA		NA		43 (43.4)		NA
Lactate dehydrogenase, U/L (IQR)	–	NA	–	NA	52	601.5 (334, 1170)	–	NA
Bilirubin, mg/dL (IQR)	–	NA	–	NA	67	1 (0.6, 2.1)	–	NA
Aspartate aminotransferase, U/L (IQR)	–	NA	–	NA	88	282 (129, 468)	–	NA
Alanine aminotransferase, U/L (IQR)	–	NA	–	NA	84	231 (137, 382)	–	NA
Creatinine,mg/dL (IQR)	–	NA	–	NA	77	0.8 (0.7, 1.0)	-	NA
Platelet count, 10^9/L (IQR)	–	NA	–	NA	90	58.5 (37.2, 89.6)	–	NA
Birthweight, grams (IQR)	127	3060 (2400, 3462)	159	3295 (3025, 3600)	83	1955 (1238, 2728)	–	NA
Fetal growth restriction (%)	135	16 (11.9)	169	10 (5.9)	89	10 (11.2)	–	NA
Gestational diabetes (%)	141	70 (49.6)	163	66 (40.5)	90	7 (7.8)	–	NA

Estimates of single-dose and double-dose effects in mothers and babies were analyzed. We also evaluated free response models and parent-of-origin effects. A two-sided significance level of α = 0.05 was used. Rare haplotypes (< 4% frequency) were excluded due to wide confidence intervals, thus resulting in a comparison of four haplotypes in the HDP population and five haplotypes in the severe PE/HELLP syndrome population. All analyses were performed using R statistical software (Version 4.2.3).

## Results

### Participant flow

**Fig. 1:**
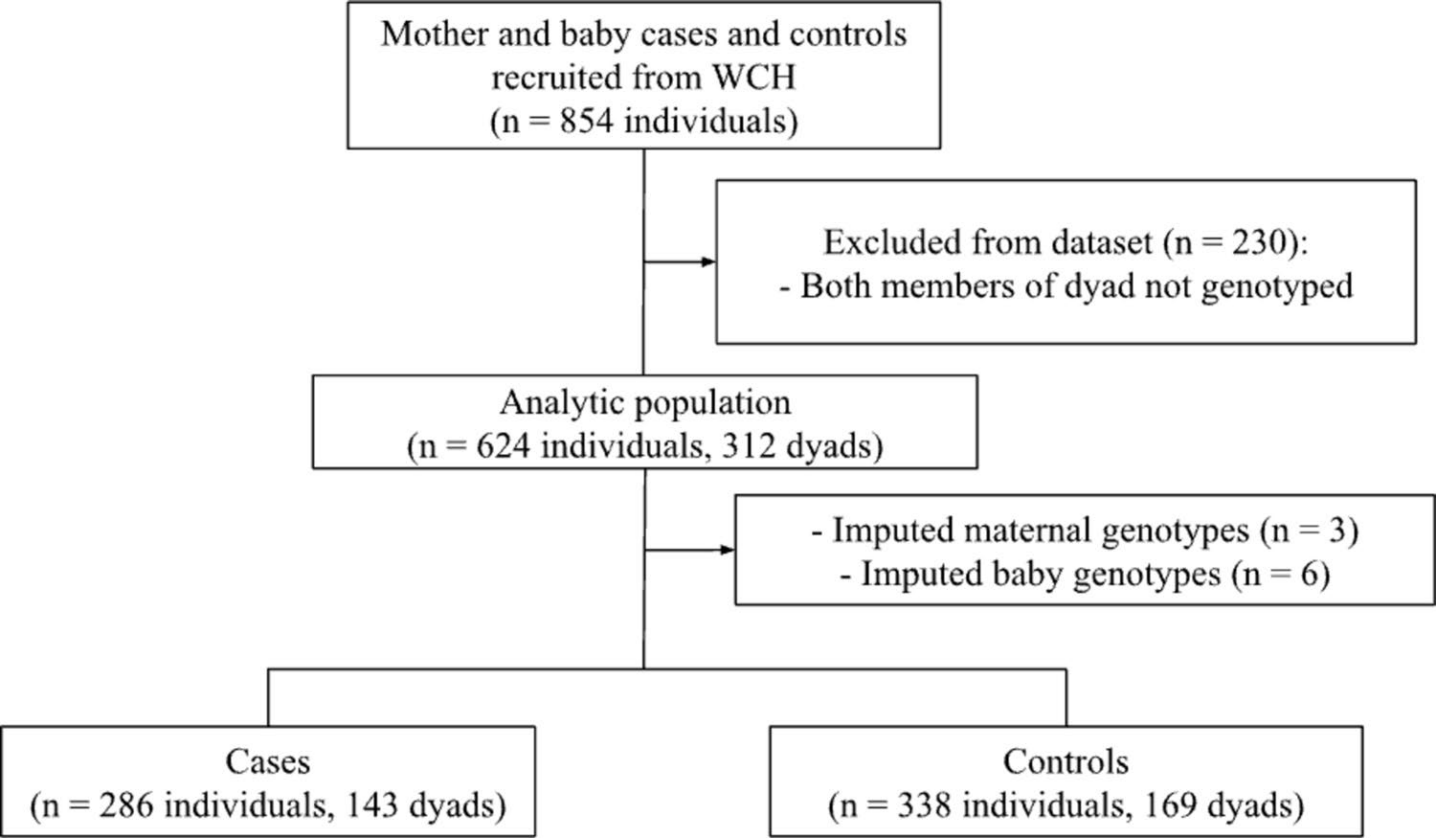
Participant flow in HDP population

HDP: Data on 854 mothers and babies were collected at baseline. A total of 230 individuals that had not been genotyped were excluded from the final analysis. Select dyads were only missing maternal genotypes (n = 3) or only missing baby genotypes (n = 6) due to failed genotyping. In this case, genotypes were imputed using Haplin’s EM algorithm. 624 mothers and babies were included in the final analytic dataset. Participant flow is illustrated in Fig. 1.

**Fig. 2:**
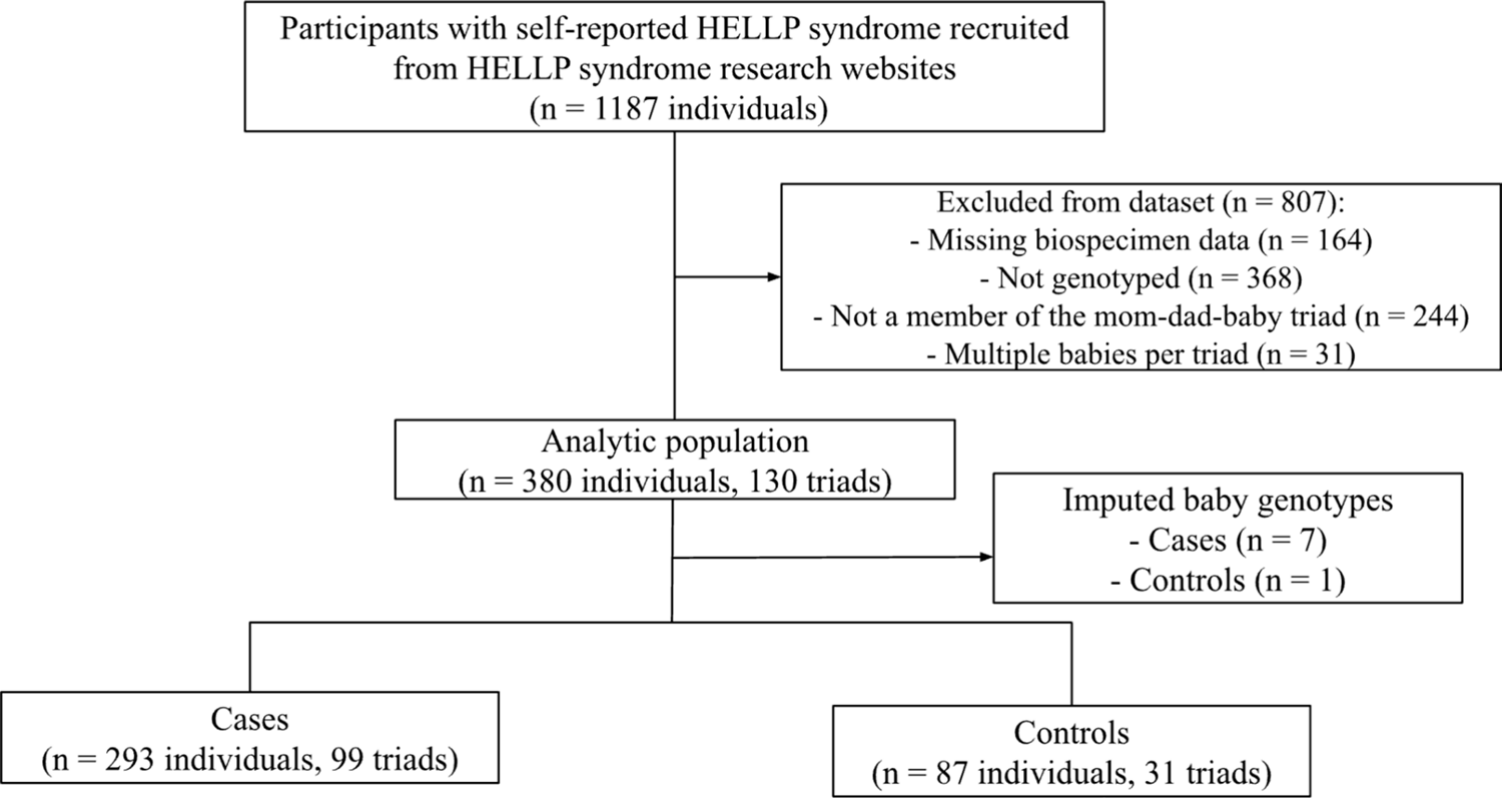
Participant flow in severe PE/HELLP syndrome population

Severe PE/HELLP: Data was collected from 1187 mothers, fathers, babies, maternal grandmothers, maternal grandfathers, sisters, or other extended family members at baseline. Exclusion criteria were: triads missing biospecimen sample (n = 164), triads that had not been genotyped (n = 368), other family members who were not mother, father, or baby (n = 244), or babies who were not the index child (n = 31). In the latter case, only 1 affected baby was randomly chosen to be kept per family via an online randomizer tool. Information on maternal grandmothers, maternal grandfathers, and sisters (n = 244) were collected in this cohort, but ultimately excluded as these family members were not a part of the mother-father-baby triad. A total of 807 individuals were excluded from the final analysis. The analytic population consisted of 380 individuals (130 triads). Figure 2 depicts the flow of subjects within this cohort.

### Maternal demographics

Medical records were reviewed for all cases in the HDP population and 92.9% of cases (n = 92) in the severe PE/HELLP syndrome population. In the severe PE/HELLP population, 56.6% of subjects were classified as severe PE (n = 56) and 43.4% were classified as HELLP syndrome (n = 43). Maternal characteristics are presented in Table 3.

HDP: The average maternal age was 27.9 ± 7.4 and 26.8 ± 7.0 among cases and controls, respectively. A majority of subjects were Hispanic (97.2% of cases, 97% of controls). A larger proportion of the cases presented as nulliparous (43%) as compared to controls (31%).

Severe PE/HELLP: Among cases and controls, the average maternal age was 31.0 ± 4.1 and 32.1 ± 4.0 respectively. Nearly all subjects were white (99% of cases, 100% of controls). Similarly, a larger proportion of the cases were nulliparous (86.5%) as compared to controls (52.2%).

### Individual SNP analysis

HDP: Data from 312 mother-baby dyads (143 cases, 169 controls) were retained for analysis. Missing genotypes were imputed for mothers (n = 3) and babies (n = 6) using Haplin’s EM algorithm. We observed a statistically significant increased risk for HDP in mothers possessing a double-dose carriage of the A allele in the SNP rs7993594 [RR = 3.52 (95% CI 1.08, 11.20), p = 0.04]. No other sFLT-1 SNP analyzed in mother and baby exhibited significant associations with HDP (Table 4).

**Table 4:** Maternal and child carriage of sFLT-1 polymorphisms and risk of HDP

SNP	Allele	Allele frequency (%)	Maternal				Child			
			Single dose RR (95% CI)	P-value	Double dose RR (95% CI)	P-value	Single dose RR (95% CI)	P-value	Double dose RR (95% CI)	P-value
rs7993594	A	9.5	0.82 (0.46, 1.45)	0.50	3.52 (1.08, 11.20)	0.04	0.98 (0.57, 1.73)	0.96	0.77 (0.10, 6.45)	0.81
rs3751395	G	24.1	1.05 (0.71, 1.58)	0.81	1.06 (0.45, 2.46)	0.89	1.29 (0.85, 1.95)	0.23	1.33 (0.58, 3.14)	0.50
rs7983774	A	16.9	1.08 (0.70, 1.69)	0.72	1.30 (0.42, 3.97)	0.65	0.76 (0.48, 1.23)	0.26	0.66 (0.19, 2.26)	0.50
rs664393	A	9.9	0.66 (0.38, 1.17)	0.15	–^1^	–^1^	0.76 (0.44, 1.34)	0.344	0.95 (0.12, 7.48)	0.95

^1^RR not estimable due to small sample size

Severe PE/HELLP: 130 mother-father-baby triad (99 cases, 31 controls) data were included in the analysis. A marginal significant protective effect was found in the child with a single-dose carriage of the A allele in SNP rs7983774 [RR = 0.59 (CI 0.36, 0.98), p = 0.05]. Other sFLT-1 SNPs examined in mother or child were not significantly associated with severe PE/HELLP syndrome risk (Table 5).

**Table 5:** Maternal and child carriage of sFLT-1 polymorphisms and risk of severe PE/HELLP syndrome

SNP	Allele	Allele frequency (%)	Maternal				Child			
			Single dose RR (95% CI)	P-value	Double dose RR (95% CI)	P-value	Single dose RR (95% CI)	P-value	Double dose RR (95% CI)	P-value
rs7993594	A	23.8	0.88 (0.54, 1.44)	0.60	0.46 (0.13, 1.69)	0.24	0.72 (0.44, 1.20)	0.21	0.38 (0.11, 1.41)	0.14
rs3751395	T	45.3	0.83 (0.49, 1.42)	0.49	1.22 (0.61, 2.49)	0.58	1.16 (0.65, 2.04)	0.61	1.35 (0.63, 2.99)	0.45
rs7983774	A	26.8	0.72 (0.44, 1.17)	0.18	0.50 (0.18, 1.45)	0.20	0.59 (0.36, 0.98)	0.05	0.50 (0.18, 1.42)	0.19
rs664393	A	11.3	0.87 (0.47, 1.64)	0.68	1.28 (0.27, 6.23)	0.75	0.98 (0.49, 1.89)	0.93	1.52 (0.32, 7.54)	0.59

### Haplotype analysis

HDP: The single dose G-T-G-G haplotype was used as a reference for both maternal and child RRs. 95 dyads were removed in the haplotype analysis due to low haplotype frequencies. In the remaining 217 dyads, no significant associations with maternal disease were found in mother or child haplotypes (Table 6).

**Table 6:** Maternal and child sFLT-1 haplotypes and risk of HDP

rs7993594 rs3751395 rs7983774 rs664393 (n = 217)									
Haplotype	Frequency (%)	Maternal				Child			
		Single dose RR (95% CI)	P-value	Double dose RR (95% CI)	P-value	Single dose RR (95% CI)	P-value	Double dose RR (95% CI)	P-value
G-g-a-a	7.0	0.96 (0.25, 3.66)	0.94	–^1^	–^1^	0.29 (0.04 2.30)	0.24	0.88 (0.04, 17)	0.93
a-g-a-G	4.3	1.35 (0.35, 5.42)	0.66	6.50 (0.50, 86.90)	0.16	0.31 (0.04, 2.51)	0.28	–^1^	–^1^
G-g-G-G	6.6	1.02 (0.27, 3.90)	0.97	–^1^	–^1^	0.59 (0.08, 4.79)	0.62	1.53 (0.12, 21.40)	0.74
G-T-G-G	81.8	REF	REF	0.93 (0.24, 3.63)	0.92	REF	REF	0.33 (0.04, 2.74)	0.32

^1^RR not estimable due to small sample size

Severe PE/HELLP: A total of 58 triads were removed from analysis due to low haplotype frequencies. An analysis of the 72 remaining triads revealed a significant increased risk in severe PE/HELLP in mothers with double-dose carriage of the G-t-G-G haplotype [RR = 4.13 (CI 1.22, 13.80), p = 0.02] (Table 7).

**Table 7:** Maternal and child sFLT-1 haplotypes and risk of severe PE/HELLP syndrome

rs7993594 rs3751395 rs7983774 rs664393 (n = 72)									
Haplotype	Frequency (%)	Maternal				Child			
		Single dose RR (95% CI)	P-value	Double dose RR (95% CI)	P-value	Single dose RR (95% CI)	P-value	Double dose RR (95% CI)	P-value
G-G-a-a	9.9	1.60 (0.53, 4.75)	0.40	4.04 (0.32, 47)	0.27	1.30 (0.40, 4.29)	0.66	2.52 (0.19, 34.90)	0.48
a-G-a-G	19.7	1.68 (0.63, 4.51)	0.29	–^1^	–^1^	0.64 (0.21, 2)	0.44	0.79 (0.07, 9.07)	0.85
G-G-G-G	23.1	2.01 (0.75, 5.46)	0.17	3.15 (0.58, 17.60)	0.19	1.16 (0.38, 3.51)	0.80	2.02 (0.35, 11.80)	0.42
a-t-G-G	6.0	1.06 (0.25, 4.39)	0.94	10.50 (0.72, 163)	0.09	1.10 (0.26, 4.42)	0.89	7.54 (0.46, 125)	0.16
G-t-G-G	40.3	REF	REF	4.13 (1.22, 13.80)	0.02	REF	REF	1.38 (0.38, 4.85)	0.63

^1^RR not estimable due to small sample size

### Parent-of-origin analysis

No parent-of-origin effects were observed in individual alleles or haplotypes in mothers or infants in the HDP or severe PE/HELLP populations.

## Discussion

Our study evaluated maternal and child genotypes and haplotypes to test for association with development of maternal disease. We found evidence of a nominally significant increased risk of HDP in mothers with a double dose, but not single dose, SNP rs7993594 and a marginally significant decreased risk in infants with a single-dose carriage of the A allele in SNP rs7983774. The haplotype analysis suggested an increased risk for severe PE/HELLP syndrome in carriers of a double dose G-t-G-G haplotype. No parent-of-origin effects were observed in the HDP and severe PE/HELLP populations.

Current literature has examined the role of other FLT1 polymorphisms on PE susceptibility. A GWAS study by McGinnis et al. had observed a significant increased risk of PE with the SNP rs4769613 in the fetal genome, with preferential inheritance of the C allele in a European population (2017). A similar study performed by Kikas et al. also showed significance in rs4769613 when stratified by placental sFLT-1 genotypes in Estonian cohorts (2020). Placental FLT1 gene expression and maternal serum sFLT-1 showed significantly higher transcript and biomarker levels in PE cases. Contrary to the results from McGinnis and Kikas, studies by Ohwaki et al. [29] and Macías-Salas et al. [22] did not find significant association of rs4769613 with predisposition to PE in non-European populations. However, both of these studies had small sample sizes which could have explained the non-significant results influenced by decreased power to detect a clinically significant genetic effect size.

Increased sFLT-1 expression has been observed in preeclamptic placentae in addition to sFLT-1 overproduction [28, 36]. Upregulated sFLT-1 levels in PE cases have been found in many similar studies [2, 4, 12, 31, 36]. sFLT-1 is released from the placenta into maternal circulation,high concentrations of sFLT-1 have been identified in both early and late-onset PE, in conjunction with diminished PlGF levels [6, 13]. This angiogenic imbalance in maternal serum has been significantly associated with placental sFLT-1 levels [8], increased sFLT-1 mRNA expression [41], and severity of preterm PE [4]. A placentally derived sFLT-1 splice variant, encoding the sFLT-1 e15a protein, is expressed in the syncytiotrophoblast and found to have a ten-fold increase in serum levels in PE cases [30]. The same study by Palmer et al. discovered that sFLT-1 e15a’s antiangiogenic properties leads to endothelial dysfunction characterized by the inhibition of endothelial cell tube formation and decreased endothelial cell migration and invasion (2015). Angiogenic imbalance by excess sFLT-1 expression and its association with PE has been well established by existing literature, however, there are no published studies on whether FLT1 genetic variants confer higher sFLT-1 expression in PE cases. Our study adds to the existing literature by introducing a genetic association within select sFLT-1 polymorphisms. An association was observed in a largely Hispanic population, contributing diversity to existing data. These results may also warrant further investigation into rs7993594 and HDP.

This study has several limitations. First, one of four SNPs (rs7993594) showed deviation from Hardy–Weinberg equilibrium (HWE). This can occur for a variety of reasons including, but not limited to: genotyping error, deletion polymorphisms, linkage disequilibrium, lack of random mating, or parental consanguinity [3, 21]. Another explanation could be the presence of maternal–fetal interactions. Secondly, the severe PE/HELLP syndrome population relied on self-report of HELLP syndrome diagnosis and only 92.9% of medical records were verified, though all cases reviewed were confirmed severe PE cases as all at least met this criteria. There may have been some misclassified cases in the severe PE/HELLP population, however, since we combined both diagnoses into the case group, this should not affect our analysis. Additionally, charts were not abstracted for the self-reported controls. If women with HDP were inadvertently included in the control group, this could have led to attenuated RR estimates toward the null. Third, our results may have been impacted by low sample size which reduces statistical power to detect the presence of true associations, especially in the severe PE/HELLP syndrome population. Additionally, small sample sizes may lead to false positives. Lastly, participants in the HDP population were largely Hispanic and the severe PE/HELLP syndrome population consisted of nearly all Caucasians. Due to the homogeneity of race/ethnicity within each cohort, our results may not be generalizable to a larger, more diverse population. We also acknowledge that, due to the lack of adjustment for multiple comparisons, Type I errors may explain some associations.

Our study also has several strengths. This was the first study to examine the four SNPs (rs7993594, rs3751395, rs7983774, rs664393) in association with HDP as well as severe PE/HELLP syndrome in parent case-dyad/triads. Additionally, the inclusion of parent data in mother-baby dyads and mother-father-baby triads allowed for the estimation of parent-of-origin effects as well as the examination of individual genetic influences within each family member. Lastly, all cases were verified PE diagnoses in the HDP population, and a majority (92.9%) of cases were verified to be diagnosed as either severe PE or HELLP syndrome through medical record abstraction in the severe PE/HELLP syndrome population.

## Conclusion

We conclude that a double-dose A allele in one FLT1 polymorphism (rs7993594), expressed maternally, appears to be associated with increased risk of HDP and a double dose of the G-t-G-G haplotype (rs7993594, rs3751395, rs7983774, rs664393) is associated with severe-spectrum PE/HELLP syndrome. Decreased risk was observed in the fetal A allele in SNP rs7983774, though this finding was of borderline significance. Future studies should examine associations utilizing a larger sample size, within a more ethnically diverse population, and with verification of diagnosis of all case participants.

## Data Availability

The data that support the findings of this study are available upon request from the corresponding author, MLW.
